# Virulent Epidemic Pneumonia in Sheep Caused by the Human Pathogen *Acinetobacter baumannii*

**DOI:** 10.3389/fmicb.2018.02616

**Published:** 2018-11-06

**Authors:** Bodo Linz, Nadia Mukhtar, Muhammad Zubair Shabbir, Israel Rivera, Yury V. Ivanov, Zarfishan Tahir, Tahir Yaqub, Eric T. Harvill

**Affiliations:** ^1^Department of Veterinary and Biomedical Sciences, The Pennsylvania State University, University Park, PA, United States; ^2^Department of Infectious Diseases, College of Veterinary Medicine, University of Georgia, Athens, GA, United States; ^3^Quality Operations Laboratory, University of Veterinary and Animal Sciences, Lahore, Pakistan

**Keywords:** *Acinetobacter baumannii*, epidemic pneumonia in sheep, Koch’s postulates, cgMLST, genome comparison, immune evasion, iron acquisition

## Abstract

The human pathogen *Acinetobacter baumannii* has emerged as a frequent cause of hospital-acquired infections, but infection of animals has rarely been observed. Here we analyzed an outbreak of epidemic pneumonia killing hundreds of sheep on a farm in Pakistan and identified *A. baumannii* as the infecting agent. A pure culture of strain AbPK1 isolated from lungs of sick animals was inoculated into healthy sheep, which subsequently developed similar disease symptoms. Bacteria re-isolated from the infected animals were shown to be identical to the inoculum, fulfilling Koch’s postulates. Comparison of the AbPK1 genome against 2283 *A. baumannii* genomes from the NCBI database revealed that AbPK1 carries genes for unusual surface structures, including a unique composition of iron acquisition genes, genes for O-antigen synthesis and sialic acid-specific acetylases of cell-surface carbohydrates that could enable immune evasion. Several of these unusual and otherwise rarely present genes were also identified in genomes of phylogenetically unrelated *A. baumannii* isolates from combat-wounded US military from Afghanistan indicating a common gene pool in this geographical region. Based on core genome MLST this virulent isolate represents a newly emerging lineage of Global Clone 2, suggesting a human source for this disease outbreak. The observed epidemic, direct transmission from sheep to sheep, which is highly unusual for *A. baumannii*, has important consequences for human and animal health. First, direct animal-to-animal transmission facilitates fast spread of pathogen and disease in the flock. Second, it may establish a stable ecological niche and subsequent spread in a new host. And third, it constitutes a serious risk of transmission of this hyper-virulent clone from sheep back to humans, which may result in emergence of contagious disease amongst humans.

## Introduction

*Acinetobacter baumannii* is an important opportunistic human pathogen that has emerged to become a predominant cause of hospital-derived infections (Dijkshoorn et al., 2007). A significant proportion of all infections by Gram-negative bacteria in ICU patients can be accounted for by *A. baumannii* (Murray and Hospenthal, 2008), and infection of combat-wounded patients constitutes an increasing threat for armed forces (Murray, 2008). The infections, which include pneumonia, meningitis, bacteremia, skin and soft tissue infections and urinary tract infections, are increasingly difficult to treat because of the ever-growing pan-resistance to antibiotics (Abbo et al., 2005). In addition to intrinsic chromosomal β-lactamase genes, many *A. baumannii* isolates possess resistance islands (RI), each of which contain multiple antibiotic resistance genes. A recent pan-genome analysis of 249 genomes from *A. baumannii* strains isolated between 1951 and 2011 revealed that strains isolated decades ago already possessed a large array of genes conferring antibiotic resistance, and showed emergence of isolates with increased number of RI insertions over time (Chan et al., 2015). However, resistance to the most advanced antibiotics such as carbapenems is generally conferred by resistance genes encoded on plasmids and transposons. The spread of these mobile elements, particularly among strains circulating within hospital environments, has led to a dramatic increase in the prevalence of multi-drug resistant (MDR) strains (Wright et al., 2014). As a result, the proportion of extensively drug-resistant *A. baumannii* strains has dramatically increased over the last 15 years, with over 60% of the isolates in the United States being reported as MDR (Sievert et al., 2013). Cases of nosocomial infection have been documented in veterinary medicine as well. Pets treated in several veterinary clinics in Europe and the Middle East were found to be colonized with *A. baumannii* (Francey et al., 2000; Endimiani et al., 2011; Zordan et al., 2011; Kuzi et al., 2016; Ewers et al., 2017). Similar to humans, *A. baumannii* were isolated from respiratory tract, urine, blood and tissues, and caused pneumonia, urinary tract infection, sepsis and cellulitis (Kuzi et al., 2016). Patterns of pulsed-field gel electrophoresis, repetitive extragenic palindromic (REP)-PCR and MLST revealed endemic occurrence and bacterial transmission within the clinics, indicating that most of the infections may have been acquired there (Zordan et al., 2011; Kuzi et al., 2016; Ewers et al., 2017). Even outside the veterinary clinics the carriage rate of 6–8% among companion animals appears to be relatively high (Belmonte et al., 2014; Pailhories et al., 2015). Based on MLST several of the animal isolates from veterinary hospitals belonged to the global clones (GC) circulating in humans and demonstrated a MDR phenotype (Ewers et al., 2017). In contrast, *A. baumannii* isolated from cattle and pigs did not belong to either of the global clones. They were not multi-drug resistant and lacked important antibiotic resistance features such as RIs and class 1 integrons suggesting that MDR *A. baumannii* found in human hospitals may not have evolved from animal strains (Hamouda et al., 2011). Instead, these observations suggest that MDR animal isolates, particularly in pets, may have been acquired from their human owners. Overall, despite a number of reports, *A. baumannii* infection of animals appears to be sporadic.

Here we analyzed an epidemic outbreak of respiratory disease among sheep and identified a newly emerging lineage of *A. baumannii* GC2 as the infecting agent. We fulfilled Koch’s postulates by inoculating the isolated bacteria into healthy animals, which developed similar disease symptoms. Whole genome analysis of the virulent isolate showed presence of an RI and of class 1 integrons, and a comparison against 2283 other *A. baumannii* genomes revealed an unusual composition of iron acquisition genes that are encoded on the genome and a plasmid, and genes for O-antigen synthesis and sialic acid-specific acetylation of cell-surface carbohydrates that could enable immune evasion.

## Materials and Methods

### Ethics Statement

The animal experiments were carried out in strict accordance with the recommendations in the Guide for the Care and Use of Laboratory Animals of the National Institutes of Health. All protocols used in this study were approved by the Ethical Research Committee of the University of Veterinary and Animal Sciences, Lahore, Pakistan (# DR/4490) and the Institutional Animal Care and Use Committee at The Pennsylvania State University, University Park, PA, United States (IACUC # 35823).

### Sample Collection

Sampling was performed from 2011 to 2012 at a Government Livestock research farm in Khizarabad in the Sargodha district in Pakistan during an epidemic outbreak of respiratory disease among sheep. The animals were anesthetized by intravenous injection of 2 mL Xylaz, and nasal swabs and broncho-alveolar lavage (BAL) were collected from 15 apparently healthy sheep and from 15 diseased animals that displayed most or all of the described symptoms (sneezing, coughing, pyrexia, abdominal breathing, weakness, see section “Results”). None of these animals had been treated with antibiotics prior to sampling. Using a sterile Stomach Tube (Curity, Tyco Healthcare, Thailand), 10 ml of sterile PBS buffer was added into the trachea, and the sheep was moved on each side several times to allow fluid to contact all parts of the lower respiratory system. Bronco-alveolar liquid (3–5 ml) was collected by aspiration with a 60 ml catheter tip disposable syringe (STAR, Jiangsu Kanghua, Medical Equipment Co., Ltd., China).

### Isolation and Identification of the Etiological Agent

Collected samples were inoculated onto Trypticase Soy, MacConkey and blood agar plates, incubated at 37°C, and examined for growth at 24 and 48 h. Morphological and preliminary biochemical tests including Gram’s staining, catalase test, oxidase test and motility test were performed for initial identification. Bacterial identification was confirmed by API 20NE (Biomerieux, Marcy l’Etoile, France). For validation, DNA was isolated from pure cultures using the Qiagen blood and tissue kit, and the 16S rDNA gene was amplified using universal primers 357-F 5′CTCCTACGGGAGGCAGCAG3′ and 1391-R 5′GACGGGCGGTGTGTACA3′ following standard protocols. PCR amplicons were purified using the QIAquick PCR purification kit (Qiagen, Valencia, CA, United States) and sequenced on an ABI3700. The sequences were analyzed using BLASTn.

### RNA Sequencing and Analysis

The BAL samples were centrifuged at 14,000 × *g* for 10 min, and total RNA was extracted from the pelleted bacteria. The RNA was quantified and quality checked with both Bioanalyzer and a Qubit fluorometer. Next-generation library preparation, library quantification and emulsion PCR were performed according to the manufacturer’s instructions (Roche Applied Science). The libraries were sequenced on a GS FLX+ instrument using GS FLX Titanium chemistry according to the manufacturer’s instructions (Roche Applied Science). GS-FLX-Titanium sequence data files (.sff) were generated using the GS amplicons software package (Roche, Branford, CT, United States). Raw sequence reads were processed in MOTHUR (Schloss et al., 2009), including quality control, trimming and screen for chimeras, in compliance with the operating manual for 454-pyrosequencing. While total RNA was isolated and sequenced, we note that only high-quality reads that aligned and mapped against the SILVA 16S rRNA database (Quast et al., 2013) were kept for further analysis, which resulted in approximately 9000 16S rRNA sequences per sample. The Operational Taxonomic Units (OTUs) were defined as sequences exhibiting greater than 97% sequence similarity. OTUs were merged to their closest taxonomic classification in METAGENASSIST (Arndt et al., 2012).

### Koch’s Postulate Study

Four healthy female sheep of 12–13 months of age were selected for an experimental infection with the previously isolated *A. baumannii* strain AbPK1. Two sheep were inoculated intra-tracheally with 5 ml containing 5×10^9^ colony forming units (CFU), two uninfected controls were kept in different boxes in the same shed. The animals were checked for symptoms at 12 h intervals and sacrificed 7 days post-inoculation. The lungs, liver, kidneys and heart of each animal were collected, and *A. baumannii* was re-isolated from each of the samples as described above.

### Whole Genome Sequencing and Analysis

The genome of the *A. baumannii* strain AbPK1 was sequenced on an Illumina MiSeq machine. Illumina TruSeq library preparation and library quantification were performed according to the manufacturer’s instructions (Illumina). Sequencing was performed as a 2 × 300 bp paired-end run with overlapping reads using Illumina MiSeq Reagent kit v3. Paired-end reads were merged using FLASH (Magoc and Salzberg, 2011) and subsequently assembled in NEWBLER (Margulies et al., 2005). Alignment of sequencing reads to the ends of all contigs was used to determine adjacent contigs. Gaps were closed by PCR and Sanger sequencing. The genes of the MLST scheme Pasteur (Diancourt et al., 2010) and the MLST scheme Oxford (Bartual et al., 2005) were extracted from the genome, and the allelic profile was determined at the *A. baumannii* MLST website^1^ sited at the University of Oxford. The core genome MLST comparison against the genomes of other ST2 isolates (MLST scheme Pasteur) was performed using the BIGSdb Genome Comparator (Jolley and Maiden, 2010) hosted at the MLST website. The resulting distance matrix was visualized in MEGA7 (Kumar et al., 2016).

The assembled genome and plasmid sequences were annotated using RAST (Aziz et al., 2008). Insertion sequence elements were detected using IS Finder (Siguier et al., 2006) and phage sequences were predicted using PHAST (Zhou et al., 2011). Resistance genes against antibiotics were analyzed in ResFinder 2.0 (Zankari et al., 2012). The identified genomic islands were verified using GIPSy (Soares et al., 2016). The whole genome comparison of AbPK1 against all available 2283 *A. baumannii* genomes (completed and draft genomes, NCBI database as of June 12, 2017) was performed using mGenomeSubtractor (Shao et al., 2010; Ou et al., 2015), an online tool for rapid *in silico* subtractive hybridization analysis of multiple bacterial genomes. All 3886 annotated protein-coding genes in the AbPK1 genome were analyzed in BLASTn searches against the other *A. baumannii* genome sequences, as were all protein-encoding genes in plasmids pAbPK1a and pAbPK1b. The identified genomic islands were verified using GIPSy (Soares et al., 2016). Presence and absence of genome and plasmid genes in (draft) genomes from all 2283 sequenced *A. baumannii* isolates was plotted using GENOMEVIZ (Ghai et al., 2004).

### Accession Numbers

The genome and plasmid sequences have been deposited in GenBank under the accession numbers CP024576 (genome AbPK1), CP024577 (plasmid-AbPK1a) and CP024578 (plasmid-AbPK1b).

## Results

### Severe Outbreak of Respiratory Disease in Sheep – Symptoms and Mortality

In 2011–2012, a severe outbreak of respiratory disease occurred in a flock of Kajli sheep in the Sargodha district of the Punjab province in Pakistan. During this 4-months long epidemic outbreak, 473 animals out of a flock of 1200 died, often within 3 days after the onset of symptoms. Symptoms included nasal discharge, coughing and pyrexia (42°C). The sheep were weak, emaciated and fast breathing, many animals showed signs of abdominal breathing. Post-mortem examination revealed anemic, swollen and congested lungs covered with white patches and with lesions on the anterior part of the lobes. In addition, the pericardial sacs were filled with a yellowish fluid.

Based on the observed symptoms, Contagious Caprine Pleuropneumonia was diagnosed, a respiratory disease common across Africa and Western Asia that is frequently caused by *Mycoplasma* species *M. capricolum* and *M. mycoides*. However, targeted antibiotics therapy with oxytetracycline and the macrolide tylosin failed to cure the diseased animals, and subsequent treatments with other antibiotics, including gentamicin, streptomycin, penicillin and sulfamethazine, were also ineffective.

### Identification of the Likely Etiological Agent

To identify the infecting agent, broncho-alveolar lavage (BAL) was sampled from 15 apparently healthy animals without symptoms and from 15 diseased animals in the flock that displayed most or all of the above symptoms. Bacteria from a small aliquot of each sample from diseased sheep formed small rounded colonies on cultivation plates with Trypticase Soy Agar. The bacteria were catalase-positive, oxidase-negative and Gram-negative coccobacilli that exhibited a non-fermenter phenotype on MacConkey agar. In contrast, such bacteria were not present in samples from healthy sheep, fulfilling Koch’s first postulate. The isolates were grown in pure culture, fulfilling Koch’s 2nd postulate, and the sequence of the 16S ribosomal RNA gene amplified with universal primers 357-F and 1391-R showed 100% identity to *A. baumannii*.

**FIGURE 1:**
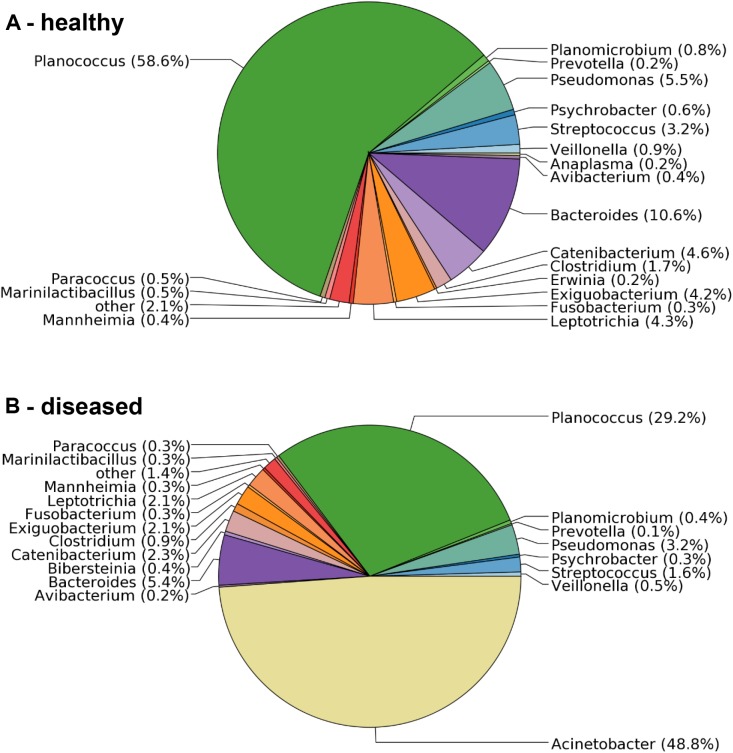
Distribution of microbial taxa in BAL samples obtained from healthy and diseased sheep. **(A)** The bronchia of healthy sheep contained bacteria from 69 genera. **(B)** Samples from diseased animals were dominated by sequencing reads assigned to the genus *Acinetobacter*, which were not found in samples from healthy sheep.

To confirm the microbiological analysis and to assess other potential etiological agents that may be present in less dominant numbers, total RNA was isolated from BAL samples collected from one healthy and three diseased sheep, and sequenced on a 454-Roche Titanium FLX sequencer. The sequencing reads were analyzed against the SILVA 16S rRNA database. They revealed a clear difference in the bacterial composition of samples obtained from apparently healthy versus diseased sheep. The sample from a healthy sheep contained 69 bacterial genera (Figure 1A), including *Planococcus* (55% of the sequencing reads), *Bacteroides* (13%) and *Pseudomonas* (6%). In contrast, while the three analyzed samples obtained from diseased animals also contained the vast majority of genera, they were dominated by *Acinetobacter* with 42.6%, 48.8% (Figure 1B) and 97.7% of the reads. All *Acinetobacter* reads were assigned to the same OTU. Since no additional taxa were identified in diseased sheep, and *Acinetobacter* was not found in samples from healthy sheep (Figure 1A), the 16S rRNA analysis confirmed the microbiology and provided additional evidence that members of this genus are the causing agent of the disease outbreak.

### Experimental Infection of Healthy Animals

Two sheep were inoculated via intra-tracheal injection with 5 × 10^9^ CFU of a liquid culture of the previously isolated *A. baumannii* strain AbPK1 to determine whether the laboratory culture would cause the same disease symptoms, fulfilling Koch’s 3rd postulate. While the two uninfected control sheep remained healthy, both inoculated sheep showed clinical symptoms within 24–48 h post-inoculation, including sneezing, coughing and abdominal breathing, indicating severe respiratory problems. The infected animals were weak and showed abnormal behavior. Post-mortem examination revealed congested nostrils and pneumonic lungs covered with large white patches and several lesions. *Acinetobacter* bacteria were re-isolated from lungs, liver and kidneys of each animal, and PCR amplification and sequencing showed that the 16S rRNA sequences of re-isolated bacteria were 100% identical to those of the inoculum, fulfilling Koch’s 4th postulate.

### MLST and Core Genome MLST Classification

**FIGURE 2:**
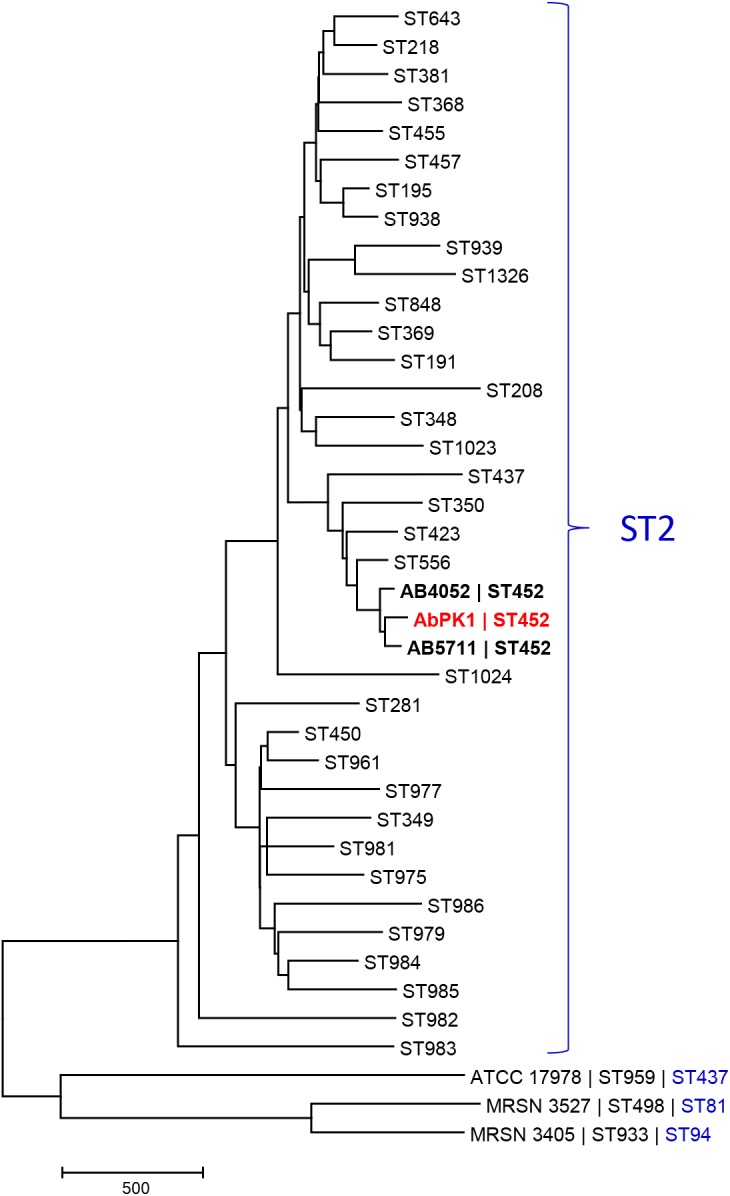
Core genome MLST-based phylogeny of global clone 2 isolates reveals that AbPK1 represents a new lineage of ST452. The minimum evolution tree displays representative genomes of the Oxford scheme STs that correspond to ST2 of MLST scheme Pasteur (=global clone 2), and genomes of three outgroup strains ATCC 17978, MRSN 3527 and MRSN 3405. Distances between taxa were calculated as the number of loci with different allele sequences. The genome of strain AbPK1 differs from its closest relatives by diverse alleles in 134 genes (AB5711) and in 156 genes (AB4052). The ST2 taxa are displayed as “Oxford scheme ST,” the three outgroup strains are displayed as “Strain name |Oxford scheme ST| Pasteur scheme ST.” ST452 in bold.

Since *A. baumannii* infection of sheep is unusual, and the high virulence and transmissibility observed within the flock unprecedented, we determined the genome sequence of strain AbPK1. The AbPK1 genome consists of a circular chromosome of 4,052,889 base pairs (bp) and two circular plasmids, pAbPK1a and pAbPK1b, of 15,113 and 79,335 bp length. Analysis of the Multi Locus Sequence Typing genes of the MLST scheme Pasteur (Diancourt et al., 2010) assigned strain AbPK1 to *A. baumannii* ST2 (allelic profile 2-2-2-2-2-2-2), indicating that it belongs to Global Clone 2 (GC2). Of the STs of the MLST scheme Oxford (Bartual et al., 2005) that correspond to ST2 of MLST scheme Pasteur, AbPK1 belongs to the rare ST452 (allelic profile 1-12-3-2-2-110-3) with only two entries in the MLST database, both of which are US military strains that were isolated from blood (strain AB5711) in 2009 and from a war wound (strain AB4052) in 2007 (Zurawski et al., 2012; Arivett et al., 2016). In order to evaluate AbPK1’s relation to other *A. baumannii* we performed a core genome MLST comparison against the genomes of other ST2 isolates using the BIGSdb Genome Comparator (Jolley and Maiden, 2010). Our analysis revealed that strain AbPK1 has a unique genotype (Figure 2). It differs from its closest ST452 relatives by diverse alleles in 134 genes (strain AB5711) and in 156 genes (strain AB4052), indicating that strain AbPK1 represents a newly emerging lineage of ST452.

### Comparative Genome Analysis of AbPK1 With 2283 Other *A. baumannii* Genomes

**FIGURE 3:**
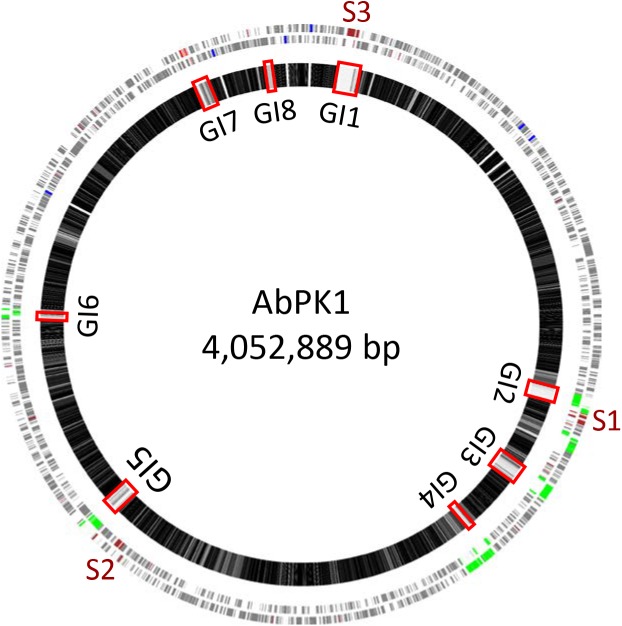
Subtractive hybridization of all 3886 CDSs of the AbPK1 genome against genomes of 2283 other *A. baumannii* isolates. Chromosome map of AbPK1 (outer two circles) with genes in forward (outermost circle) and reverse orientation. S1 to S3 – siderophore loci; brown – iron acquisition genes; red – antibiotic resistance genes; blue – ribosomal RNA; green – phage. Inner circle: Chromosome of AbPK1 with gray shade-coded genes based on the percentage of compared *A. baumannii* genomes possessing a homologous sequence. Genes present in near 100% of the genomes are shown in black, genes almost unique to AbPK1 are shown in white. Non-coding regions are shown as gaps. The Genomic Islands (GI) are marked by red rectangles.

**FIGURE 4:**
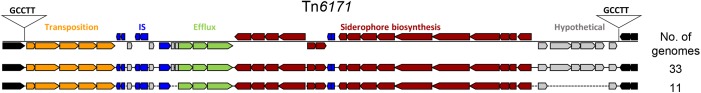
Pathogenicity Island 1 carrying siderophore cluster 3. The entire island consisting of Tn*6171* with target site duplication “GCCTT” was found present in 44 out of 2283 (1.9%) analyzed *A. baumannii* genomes. brown: siderophore biosynthesis genes; gray: hypothetical protein genes; green: efflux pump genes; blue: IS elements; orange: transposition gene cluster; black: flanking genes present in all genomes.

We used mGenomeSubtractor (Shao et al., 2010; Ou et al., 2015) to analyze presence or absence of homologs of all protein-coding genes (CDSs) in all 2283 available *A. baumannii* genomes (NCBI database as of June 12, 2017). The AbPK1 genome contains large stretches of genomic DNA present in all genomes (black boxes in Figure 3), and eight genomic islands (GI1–GI8) with clusters of genes rarely present in the other genomes (Figure 3 and Table 1). GI7 appears to be AbaR4-type resistance island (RI) inserted within the *comM* gene (Supplementary Figure S1). In addition, the genome of AbPK1 contains two putative pathogenicity islands (PAIs). GI1 (PAI1), which consists of transposon Tn*6171* (Hamidian et al., 2015), harbors a 16-gene cluster encoding proteins involved in the biosynthesis of siderophores, a 3-gene SunT-like bacteriocin exporter and a five-gene transposon Tn7 transposition gene locus (Figures 3, 4 and Table 1). GI9, another likely PAI, contains an O-antigen synthesis locus with 12 genes, which replaced part of a K capsule synthesis locus (Figure 3 and Table 1). The remaining GIs contain many genes encoding phage-like proteins of unknown functions. In addition, GI2, GI3, and GI5 carry genes for sialic acid-specific acetyltransferases.

**Table 1:** Genomic Islands (GI) identified within the genome of strain AbPK1.

Region	Type	Genome coordinates	Size (kb)	GC%	Features
GI1	PAI	92,671–143,870	51.2	37.6	Transposon Tn*6171* encompassing the entire island, containing a siderophore synthesis gene cluster (16 genes), an ABC-type bacteriocin/antibiotic exporter, IS*Aba1*, IS*Aba12* and *tnsABCDE* transposon Tn7 transposition cluster. Island flanked by TSD CTTGG
GI2	Phage	1,172,836–1,209,618	36.8	41.3	Mostly phage proteins, Sialic acid acetyltransferase 1; Island flanked by TSD ACTATAG
GI3	Phage	1,377,571–1,419,735	42.2	39.9	Mostly phage proteins, Sialic acid acetyltransferase 2
GI4	Phage	1,564,818–1,582,283	17.5	38.7	Mostly phage proteins, IS*Aba1*
GI5	Phage	2,532,268–2,567,203	34.9	40.1	Mostly phage proteins, Sialic acid acetyltransferase 3; Island flanked by TSD AAAAAGCGCTCAATCTAGAGCG
GI6	Phage	3,052,809–3,076,384	23.6	37.5	Mostly phage proteins, IS*Aba19*
GI7	RI	3,780,789–3,815,504	34.7	39.8	Resistance island containing streptomycin resistance genes *strA* and *strB*, aminoglycoside O-phosphotransferase gene *aph(3’)-VIb*, sulphonamide resistance gene *sul2*, transposon Tn*1213* with class A β-lactamase integrated in IS element IS*Pa14*, genes encoding transposition module TniABC; Island flanked by TSD CTTGG
GI8	PAI	3,964,283–3,976,553	12.3	32.1	A 13-gene O-antigen cluster inserted in the K capsule locus.


*TSD – Target site duplication.*

### AbPK1 Genes Associated With Antimicrobial Resistance

**FIGURE 5:**
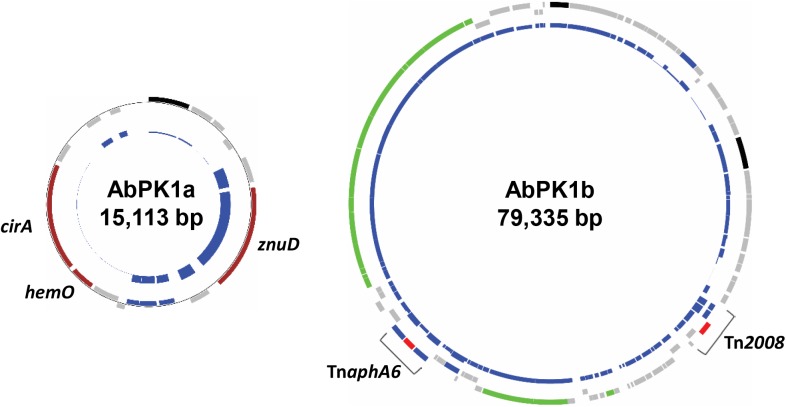
Plasmids in strain AbPK1 containing iron acquisition genes (AbPK1a), transposons and a conjugation gene cluster (AbPK1b). Outer circles: protein-encoding genes in forward (outer rectangles) and reverse (inner rectangles) orientation color-coded by function; brown – iron acquisition; gray – hypothetical protein; black – plasmid replication; green – conjugation; blue – IS elements; red – antibiotic resistance. Inner circles: Relative frequency of gene presence in 2283 sequenced *A. baumannii* isolates. While the *znuD* gene was present in 56% of the analyzed genomes, *cirA* and *hemO* were found in only 26 genomes (1.1%).

ResFinder (Zankari et al., 2012) analysis showed that the genome of strain AbPK1 possesses four different aminoglycoside resistance-encoding genes [*aac(3)-Ia, strA, strB*, and *aph(3′)-VIb*] and four different β-lactamase genes (*bla*_ADC-25_, *bla*_OXA-66_, *bla*_PER-1_, and *bla*_OXA-23_). The aminoglycoside 3’-phosphotransferase gene *aph(3’)-VIb*, streptomycin 3’-kinase genes *strA* and *strB*, β-lactam resistance gene *bla*_PER-1_, as well as gene *sul2* that provides resistance against sulfonamides are located on the RI (Table 1 and Supplementary Figure S1). All of the antibiotic resistance genes are associated with insertion sequence (IS) elements, with the exception of *bla*_OXA-66_, and several are part of transposons. The AbPK1 genome contains 14 copies of IS*Aba1*, 17 copies of IS*Aba19* and 2 copies of IS*26*, as well as single copies of five other IS elements. Transposon Tn*1213* carrying a *bla*_PER-1_ β-lactamase is part of RI. Transposon Tn*2008*, which contains carbapenems resistance gene *bla*_OXA-23_, is present as two identical copies in the genome and a further copy on plasmid pAbPK1a (Supplementary Figure S1). This plasmid further contains transposon Tn*aphA6* with two IS*Aba125* elements flanking phosphotransferase gene *aph(3’)-VIa* (Figure 5).

In addition to these acquired resistance genes, a detailed analysis of MDR-related genetic determinants revealed the presence of at least five sets of RND (resistance-nodulation-division) family efflux pumps (AdeABC, AdeFGH, AdeIJK, CzcABC, MacABC). Notably, RND locus *macABC* encodes a macrolide-specific efflux pump (Kobayashi et al., 2001), and efflux pumps encoded by the *adeABC* (Magnet et al., 2001), *adeFGH* (Coyne et al., 2010) and *adeIJK* (Damier-Piolle et al., 2008) genes are known to increase resistance to multiple antibiotics. In addition, strain AbPK1 possesses AcrAB-like and EmrAB-like RND efflux pumps, multidrug resistance transporters of the Bcr/CflA family, and MATE (Multidrug and toxic compound extrusion) efflux pumps (Supplementary Table S1).

### Genomic Properties That May Determine Host Specificity and Virulence

Similar to other *A. baumannii* genomes, the AbPK1 genome possesses the genes encoding important virulence factors such as the adhesin OmpA, phospholipase C and D, serum resistance protein PgpG, and factors shown to be involved in biofilm formation (Bap, Csu fimbriae, PgaABCD, efflux pump AdeFGH) and LPS biosynthesis genes. However, a large part of the K capsule locus was replaced by an O-antigen encoding gene cluster (GI8, Figure 3 and Table 1), which is also present in 495 of the 2283 (21.7%) analyzed *A. baumannii* genomes (NCBI database as of June 12, 2017). In addition to O-antigen, the sialic acid-specific acetyltransferases encoded on GI2, GI3, and GI5 (Table 1) may have played a role in immune evasion by altering polysaccharide structures on the bacterial surface. These acetyltransferase genes are present in only 8 (0.35%), 152 (6.7%), and 481 (21%) other *A. baumannii* genomes, respectively.

Iron is critical for survival of bacterial pathogens during colonization, and proteins involved in iron acquisition from their mammalian hosts have been regarded as important virulence factors (Wilks and Burkhard, 2007). The AbPK1 genome contains three large siderophore encoding loci (S1–S3 in Figure 3), two of which have previously been described as part of the *A. baumannii* core genome (Chan et al., 2015), namely the acinetobactin locus (S1, A1S_2372 to A1S_2392 in the genome of strain ATCC 17978) and siderophore locus 2 (A1S_1647 to A1S_1657), but locus 2 is likely not functional because of genes (orthologs of A1S_1647 and A1S_1654) disrupted by insertion of IS elements. Siderophore cluster 3 (A1S_2581 to A1S_2562), a 16-gene cluster for siderophore biosynthesis and transport on PAI1 known as transposon Tn*6171* (Figure 4), is only present in 44 out of 2283 other *A. baumannii* genomes. Similar to genomes of recently sequenced isolates MRSN 3405, MRSN 3527, MRSN 3942, MRSN 4106, and AB4052 from US military patients (Lesho et al., 2013; Chan et al., 2015; Arivett et al., 2016), transposon Tn*6171* is inserted in AbPK1 at a different chromosomal location (between A1S_3398 and A1S_3396) than in the genome of strain ATCC 17978. Additional iron acquisition proteins are encoded on plasmid pAbPK1a (Figure 5), including a homolog of zinc- and heme-uptake protein ZnuD, which was frequently present in other *A. baumannii* genomes (in 56%). In contrast, genes encoding a heme/hemoglobin receptor CirA-like protein and heme oxygenase HemO were rarely present, they were found in only 26 out of the 2283 (1.1%) other *A. baumannii* (draft) genomes, 11 of which also possess siderophore locus 3. It is tempting to speculate that the vastly different iron acquisition systems may have contributed to strains AbPK1’s ability to cause an epidemic outbreak of pneumonia in sheep.

## Discussion

Sheep and goats are important livestock in many regions of the world. Sheep are known to be susceptible to a variety of bacterial infections, including Caseous Lymphadenitis (caused by *Corynebacterium pseudotuberculosis*) and Contagious caprine pleuropneumonia (caused by *M. capricolum* and *M. mycoides*). In this study, we identified *A. baumannii* as the causative agent of an epidemic outbreak of pneumonia and successfully fulfilled the criteria that were established by Robert Koch to determine the causative agent of a particular disease (Evans, 1976; Falkow, 2004). The pathogen was identified in samples from diseased, but not from healthy sheep (postulate I, Figure 1). The pathogen was isolated from diseased animals and grown in pure culture (postulate II) and caused similar symptoms when inoculated into healthy animals (postulate III). And finally, the pathogen was re-isolated from the inoculated animals and shown to be identical to the original isolate (postulate IV).

Multidrug resistance of *A. baumannii* has been an increasing health care problem, and strain AbPK1 that caused high mortality in Kajli sheep was resistant against treatment with broad variety of antibiotics, including macrolides (tylosin), penicillin, aminoglycosides (gentamicin, streptomycin), and sulphonamides (sulfamethazine). In addition to specific, acquired resistance genes such as carbapenemase gene *bla*_OXA-23_, efflux pumps decrease *A. baumannii* susceptibility to antibiotics by pumping antimicrobials out of the bacterial cell (Magnet et al., 2001; Damier-Piolle et al., 2008; Coyne et al., 2010). Similar to many other isolates (Abbo et al., 2005), AbPK1 possesses antibiotic resistance genes on a resistance island (GI7), on transposons and on plasmids, suggesting that they may have been acquired via horizontal gene transfer (HGT). Rapid acquisition and/or loss of mobile elements is a major player in the generation of genetic diversity. In *A. baumannii* variation in gene content appears to occur more frequently and faster than the accumulation of SNPs (Grana-Miraglia et al., 2017). Frequent HGT has been observed both at a local scale during expansion of a single clone (Grana-Miraglia et al., 2017), as well as globally across different lineages (Chan et al., 2015), such as the presence of the siderophore gene cluster 3 in strain AbPK1 and in phylogenetically unrelated isolates from US military patients.

The extraordinary epidemic caused by this strain may be related to its relatively unique set of genes. Siderophores are iron-sequestration systems utilized by bacterial pathogens to acquire the scarcely available iron in mammalian host environments. While siderophore clusters 1 and 2 are ubiquitously present among *A. baumannii* genomes, the prevalence of siderophore locus 3 (Tn*6171*; Figures 3, 4) is very low. Tn*6171* is present in the genome of strain ATCC 17978 (Smith et al., 2007), albeit at a different chromosomal location than in the genomes of strain AbPK1 and of five recent isolates from US military patients (Lesho et al., 2013; Chan et al., 2015; Arivett et al., 2016). Based on (cg)MLST, strains AbPK1 (MLST Pasteur ST2, allele summary 2-2-2-2-2-2-2), ATCC 17978 (ST437, 3-2-2-2-30-4-28) and the US military strains MRSN 3527 (ST81, 1-1-1-1-5-1-2) and MRSN 3405 (ST94, 1-2-2-1-5-1-1) belong to four different evolutionary lineages of *A. baumannii* (Figure 2) implying multiple independent events involving acquisition of this genomic island. Strain ATCC 17978 was collected in 1951, whereas the US military strains were isolated in 2011 from combat-wounded patients in Afghanistan (Lesho et al., 2013), and AbPK1 was isolated in 2012 from sheep in the neighboring country, Pakistan. Acquisition of Tn*6171* in phylogenetically unrelated modern isolates indicates re-emergence of a “novel” iron scavenging mechanism from an older gene pool. The expression of siderophore cluster 3 was shown to be highly up-regulated under iron limiting conditions (Eijkelkamp et al., 2011), and expression of different types of iron acquisition proteins, including plasmid-born, may help to circumvent host defense mechanisms such as siderophore inhibitors (Flo et al., 2004; Wilson et al., 2016). Likewise, modifications of bacterial surface polysaccharides may have played a major role in immune evasion. Sialic acid-specific acetyltransferases alter capsular polysaccharides by acetylation of sialic acid residues, which affects the antigenicity and the chemical properties of the capsule. Acetylation can prevent activation of sialic acid-binding immunoglobulin superfamily lectins and activation of neutrophils (Carlin et al., 2009; Weiman et al., 2009), as well as restrict deposition of complement on the bacterial surface. Thus, inhibition of important innate immune functions may have been crucial for the spread of *A. baumannii* in sheep.

While infection with *A. baumannii* has been described for horses and for pet cats and dogs, particularly nosocomial infection in veterinary hospital settings (Endimiani et al., 2011; Zordan et al., 2011; Belmonte et al., 2014), an epidemic outbreak of *A. baumannii*-induced pneumonia among sheep is unprecedented. This severe *A. baumannii* outbreak raises questions about likely sources as well as about the transmission mode during this plague. Since *A. baumannii* strains have been found in various environments (Berlau et al., 1999; Sarma et al., 2004; Dijkshoorn et al., 2007; Peleg et al., 2008; Choi et al., 2012), acquisition of the bacteria from soil, food or water seems conceivable. However, strain AbPK1 belongs to GC2, which argues for initial acquisition from a human, possibly asymptomatic, carrier. During the epidemic, direct transmission from sheep to sheep appears likely because of the rapid progression of the epidemic and the observed symptoms such as coughing, which can effectively disseminate the pathogen in airborne droplets. A direct animal-to-animal transmission cycle of the pathogen, which is uncommon for *A. baumannii*, has important implications for animal and human health. First, epidemic spread of the pathogen by direct transmission facilitates rapid dissemination and thus progression of the disease among susceptible domestic animals with a potentially high economic burden for the farmer. Second, airborne epidemic spread constitutes a serious risk of transmission of this hyper-virulent clone from sheep back to humans, especially to farm workers with close proximity to the animals. Fortunately, neither spread to other animal species nor spread to humans has been observed during the outbreak. And third, direct transmission can establish a complete cycle in animals that were previously transient hosts or possibly a dead end, creating a stable ecological niche in a new host species, which then can in turn result in human infection from an infectious source of contagious disease in sheep. Afghanistan and Pakistan are neighboring countries, and epidemic spread of a newly emerging pathogen clone, that shares unusual genomic properties with isolates from combat-wounded US military, emphasizes the risk of worldwide dissemination of highly virulent isolates.

## Author Contributions

BL, NM, MZS, TY, and ETH designed the study and experiments. BL, NM, MZS, ZT, and TY performed the experiments. BL, NM, MZS, IR, and YVI analyzed the data. BL wrote the paper.

## Conflict of Interest Statement

The authors declare that the research was conducted in the absence of any commercial or financial relationships that could be construed as a potential conflict of interest.

## Footnotes

1 http://pubmlst.org/abaumannii/

## References

[B1] Abbo A. Navon-Venezia S. Hammer-Muntz O. Krichali T. Siegman-Igra Y. Carmeli Y. (2005). Multidrug-resistant *Acinetobacter baumannii*. *Emerg. Infect. Dis.* 11 22–29. 10.3201/eid1101.040001 15705318 PMC3294361

[B2] Arivett B. A. Ream D. C. Fiester S. E. Kidane D. Actis L. A. (2016). Draft genome sequences of *Acinetobacter baumannii* isolates from wounded military personnel. *Genome Announc* 4:e00773-16. 10.1128/genomeA.00773-16 27563036 PMC5000820

[B3] Arndt D. Xia J. Liu Y. Zhou Y. Guo A. C. Cruz J. A. (2012). METAGENassist: a comprehensive web server for comparative metagenomics. *Nucleic Acids Res.* 40(Web Server issue), W88–W95. 10.1093/nar/gks497 22645318 PMC3394294

[B4] Aziz R. K. Bartels D. Best A. A. DeJongh M. Disz T. Edwards R. A. (2008). The RAST Server: rapid annotations using subsystems technology. *BMC Genomics* 9:75. 10.1186/1471-2164-9-75 18261238 PMC2265698

[B5] Bartual S. G. Seifert H. Hippler C. Luzon M. A. Wisplinghoff H. Rodriguez-Valera F. (2005). Development of a multilocus sequence typing scheme for characterization of clinical isolates of *Acinetobacter baumannii*. *J. Clin. Microbiol.* 43 4382–4390. 10.1128/JCM.43.9.4382-4390.2005 16145081 PMC1234098

[B6] Belmonte O. Pailhories H. Kempf M. Gaultier M. P. Lemarie C. Ramont C. (2014). High prevalence of closely-related *Acinetobacter baumannii* in pets according to a multicentre study in veterinary clinics, Reunion Island. *Vet. Microbiol.* 170 446–450. 10.1016/j.vetmic.2014.01.042 24613079

[B7] Berlau J. Aucken H. M. Houang E. Pitt T. L. (1999). Isolation of *Acinetobacter* spp. including *A. baumannii* from vegetables: implications for hospital-acquired infections. *J. Hosp. Infect.* 42 201–204. 10.1053/jhin.1999.0602 10439992

[B8] Carlin A. F. Uchiyama S. Chang Y. C. Lewis A. L. Nizet V. Varki A. (2009). Molecular mimicry of host sialylated glycans allows a bacterial pathogen to engage neutrophil Siglec-9 and dampen the innate immune response. *Blood* 113 3333–3336. 10.1182/blood-2008-11-187302 19196661 PMC2665898

[B9] Chan A. P. Sutton G. DePew J. Krishnakumar R. Choi Y. Huang X. Z. (2015). A novel method of consensus pan-chromosome assembly and large-scale comparative analysis reveal the highly flexible pan-genome of *Acinetobacter baumannii*. *Genome Biol.* 16:143. 10.1186/s13059-015-0701-6 26195261 PMC4507327

[B10] Choi J. Y. Kim Y. Ko E. A. Park Y. K. Jheong W. H. Ko G. (2012). *Acinetobacter* species isolates from a range of environments: species survey and observations of antimicrobial resistance. *Diagn. Microbiol. Infect. Dis.* 74 177–180. 10.1016/j.diagmicrobio.2012.06.023 22902160

[B11] Coyne S. Rosenfeld N. Lambert T. Courvalin P. Perichon B. (2010). Overexpression of resistance-nodulation-cell division pump AdeFGH confers multidrug resistance in *Acinetobacter baumannii*. *Antimicrob. Agents Chemother.* 54 4389–4393. 10.1128/AAC.00155-10 20696879 PMC2944555

[B12] Damier-Piolle L. Magnet S. Bremont S. Lambert T. Courvalin P. (2008). AdeIJK, a resistance-nodulation-cell division pump effluxing multiple antibiotics in *Acinetobacter baumannii*. *Antimicrob. Agents Chemother.* 52 557–562. 10.1128/AAC.00732-07 18086852 PMC2224764

[B13] Diancourt L. Passet V. Nemec A. Dijkshoorn L. Brisse S. (2010). The population structure of *Acinetobacter baumannii*: expanding multiresistant clones from an ancestral susceptible genetic pool. *PLoS One* 5:e10034. 10.1371/journal.pone.0010034 20383326 PMC2850921

[B14] Dijkshoorn L. Nemec A. Seifert H. (2007). An increasing threat in hospitals: multidrug-resistant *Acinetobacter baumannii*. *Nat. Rev. Microbiol.* 5 939–951. 10.1038/nrmicro1789 18007677

[B15] Eijkelkamp B. A. Hassan K. A. Paulsen I. T. Brown M. H. (2011). Investigation of the human pathogen *Acinetobacter baumannii* under iron limiting conditions. *BMC Genomics* 12:126. 10.1186/1471-2164-12-126 21342532 PMC3055841

[B16] Endimiani A. Hujer K. M. Hujer A. M. Bertschy I. Rossano A. Koch C. (2011). *Acinetobacter baumannii* isolates from pets and horses in Switzerland: molecular characterization and clinical data. *J. Antimicrob. Chemother.* 66 2248–2254. 10.1093/jac/dkr289 21733964 PMC3172040

[B17] Evans A. S. (1976). Causation and disease: the Henle-Koch postulates revisited. *Yale J. Biol. Med.* 49 175–195. 782050 PMC2595276

[B18] Ewers C. Klotz P. Leidner U. Stamm I. Prenger-Berninghoff E. Gottig S. (2017). OXA-23 and ISAba1-OXA-66 class D beta-lactamases in *Acinetobacter baumannii* isolates from companion animals. *Int. J. Antimicrob. Agents* 49 37–44. 10.1016/j.ijantimicag.2016.09.033 27890443

[B19] Falkow S. (2004). Molecular Koch’s postulates applied to bacterial pathogenicity–a personal recollection 15 years later. *Nat. Rev. Microbiol.* 2 67–72. 10.1038/nrmicro799 15035010

[B20] Flo T. H. Smith K. D. Sato S. Rodriguez D. J. Holmes M. A. Strong R. K. (2004). Lipocalin 2 mediates an innate immune response to bacterial infection by sequestrating iron. *Nature* 432 917–921. 10.1038/nature03104 15531878

[B21] Francey T. Gaschen F. Nicolet J. Burnens A. P. (2000). The role of *Acinetobacter baumannii* as a nosocomial pathogen for dogs and cats in an intensive care unit. *J. Vet. Int. Med.* 14 177–183. 10.1111/j.1939-1676.2000.tb02233.x 10772490

[B22] Ghai R. Hain T. Chakraborty T. (2004). GenomeViz: visualizing microbial genomes. *BMC Bioinformatics* 5:198. 10.1186/1471-2105-5-198 15601465 PMC544189

[B23] Grana-Miraglia L. Lozano L. F. Velazquez C. Volkow-Fernandez P. Perez-Oseguera A. Cevallos M. A. (2017). Rapid gene turnover as a significant source of genetic variation in a recently seeded population of a healthcare-associated pathogen. *Front. Microbiol.* 8:1817. 10.3389/fmicb.2017.01817 28979253 PMC5611417

[B24] Hamidian M. Hawkey J. Holt K. E. Hall R. M. (2015). Genome sequence of *acinetobacter baumannii* strain d36, an antibiotic-resistant isolate from lineage 2 of global clone 1. *Genome Announc.* 3:e01478-15. 10.1128/genomeA.01478-15 26679588 PMC4683233

[B25] Hamouda A. Findlay J. Al Hassan L. Amyes S. G. (2011). Epidemiology of *Acinetobacter baumannii* of animal origin. *Int. J. Antimicrob. Agents* 38 314–318. 10.1016/j.ijantimicag.2011.06.007 21831604

[B26] Jolley K. A. Maiden M. C. (2010). BIGSdb: scalable analysis of bacterial genome variation at the population level. *BMC Bioinformatics* 11:595. 10.1186/1471-2105-11-595 21143983 PMC3004885

[B27] Kobayashi N. Nishino K. Yamaguchi A. (2001). Novel macrolide-specific ABC-type efflux transporter in *Escherichia coli*. *J. Bacteriol.* 183 5639–5644. 11544226 10.1128/JB.183.19.5639-5644.2001 PMC95455

[B28] Kumar S. Stecher G. Tamura K. (2016). MEGA7: molecular evolutionary genetics analysis version 7.0 for Bigger Datasets. *Mol. Biol. Evol.* 33 1870–1874. 10.1093/molbev/msw054 27004904 PMC8210823

[B29] Kuzi S. Blum S. E. Kahane N. Adler A. Hussein O. Segev G. (2016). Multi-drug-resistant *Acinetobacter* calcoaceticus-*Acinetobacter baumannii* complex infection outbreak in dogs and cats in a veterinary hospital. *J. Small Anim. Pract.* 57 617–625. 10.1111/jsap.12555 27709647

[B30] Lesho E. Yoon E. J. McGann P. Snesrud E. Kwak Y. Milillo M. (2013). Emergence of colistin-resistance in extremely drug-resistant *Acinetobacter baumannii* containing a novel pmrCAB operon during colistin therapy of wound infections. *J. Infect. Dis.* 208 1142–1151. 10.1093/infdis/jit293 23812239

[B31] Magnet S. Courvalin P. Lambert T. (2001). Resistance-nodulation-cell division-type efflux pump involved in aminoglycoside resistance in *Acinetobacter baumannii* strain BM4454. *Antimicrob. Agents Chemother.* 45 3375–3380. 10.1128/AAC.45.12.3375-3380.2001 11709311 PMC90840

[B32] Magoc T. Salzberg S. L. (2011). FLASH: fast length adjustment of short reads to improve genome assemblies. *Bioinformatics* 27 2957–2963. 10.1093/bioinformatics/btr507 21903629 PMC3198573

[B33] Margulies M. Egholm M. Altman W. E. Attiya S. Bader J. S. Bemben L. A. (2005). Genome sequencing in microfabricated high-density picolitre reactors. *Nature* 437 376–380. 10.1038/nature03959 16056220 PMC1464427

[B34] Murray C. K. (2008). Epidemiology of infections associated with combat-related injuries in Iraq and Afghanistan. *J. Trauma* 64 3(Suppl.), S232–S238. 10.1097/TA.0b013e318163c3f5 18316967

[B35] Murray C. K. Hospenthal D. R. (2008). Acinetobacter infection in the ICU. *Critc. Care Clin.* 24 237–248, vii. 10.1016/j.ccc.2007.12.005 18361943

[B36] Ou H. Y. Kuang S. N. He X. Molgora B. M. Ewing P. J. Deng Z. (2015). Complete genome sequence of hypervirulent and outbreak-associated *Acinetobacter baumannii* strain LAC-4: epidemiology, resistance genetic determinants and potential virulence factors. *Sci. Rep.* 5:8643. 10.1038/srep08643 25728466 PMC4345345

[B37] Pailhories H. Belmonte O. Kempf M. Lemarie C. Cuziat J. Quinqueneau C. (2015). Diversity of *Acinetobacter baumannii* strains isolated in humans, companion animals, and the environment in Reunion Island: an exploratory study. *Int. J. Infect. Dis.* 37 64–69. 10.1016/j.ijid.2015.05.012 26093214

[B38] Peleg A. Y. Seifert H. Paterson D. L. (2008). *Acinetobacter baumannii*: emergence of a successful pathogen. *Clin. Microbiol. Rev.* 21 538–582. 10.1128/CMR.00058-07 18625687 PMC2493088

[B39] Quast C. Pruesse E. Yilmaz P. Gerken J. Schweer T. Yarza P. (2013). The SILVA ribosomal RNA gene database project: improved data processing and web-based tools. *Nucleic Acids Res.* 41(Database issue), D590–D596. 10.1093/nar/gks1219 23193283 PMC3531112

[B40] Sarma P. M. Bhattacharya D. Krishnan S. Lal B. (2004). Assessment of intra-species diversity among strains of Acinetobacter baumannii isolated from sites contaminated with petroleum hydrocarbons. *Can. J. Microbiol.* 50 405–414. 10.1139/w04-018 15284886

[B41] Schloss P. D. Westcott S. L. Ryabin T. Hall J. R. Hartmann M. Hollister E. B. (2009). Introducing mothur: open-source, platform-independent, community-supported software for describing and comparing microbial communities. *Appl. Environ. Microbiol.* 75 7537–7541. 10.1128/AEM.01541-09 19801464 PMC2786419

[B42] Shao Y. He X. Harrison E. M. Tai C. Ou H. Y. Rajakumar K. (2010). mGenomeSubtractor: a web-based tool for parallel in silico subtractive hybridization analysis of multiple bacterial genomes. *Nucleic Acids Res.* 38(Web Server issue), W194–W200. 10.1093/nar/gkq326 20435682 PMC2896100

[B43] Sievert D. M. Ricks P. Edwards J. R. Schneider A. Patel J. Srinivasan A. (2013). Antimicrobial-resistant pathogens associated with healthcare-associated infections: summary of data reported to the National Healthcare Safety Network at the Centers for Disease Control and Prevention, 2009-2010. *Infect. Control Hosp. Epidemiol.* 34 1–14. 10.1086/668770 23221186

[B44] Siguier P. Perochon J. Lestrade L. Mahillon J. Chandler M. (2006). ISfinder: the reference centre for bacterial insertion sequences. *Nucleic Acids Res.* 34(Database issue), D32–D36. 10.1093/nar/gkj014 16381877 PMC1347377

[B45] Smith M. G. Gianoulis T. A. Pukatzki S. Mekalanos J. J. Ornston L. N. Gerstein M. (2007). New insights into *Acinetobacter baumannii* pathogenesis revealed by high-density pyrosequencing and transposon mutagenesis. *Genes Dev.* 21 601–614. 10.1101/gad.1510307 17344419 PMC1820901

[B46] Soares S. C. Geyik H. Ramos R. T. de Sa P. H. Barbosa E. G. Baumbach J. (2016). GIPSy: genomic island prediction software. *J. Biotechnol.* 232 2–11. 10.1016/j.jbiotec.2015.09.008 26376473

[B47] Weiman S. Dahesh S. Carlin A. F. Varki A. Nizet V. Lewis A. L. (2009). Genetic and biochemical modulation of sialic acid O-acetylation on group B *Streptococcus*: phenotypic and functional impact. *Glycobiology* 19 1204–1213. 10.1093/glycob/cwp111 19643844 PMC2757575

[B48] Wilks A. Burkhard K. A. (2007). Heme and virulence: how bacterial pathogens regulate, transport and utilize heme. *Nat. Prod. Rep.* 24 511–522. 10.1039/b604193k 17534527

[B49] Wilson B. R. Bogdan A. R. Miyazawa M. Hashimoto K. Tsuji Y. (2016). Siderophores in iron metabolism: from mechanism to therapy potential. *Trends Mol. Med.* 22 1077–1090. 10.1016/j.molmed.2016.10.005 27825668 PMC5135587

[B50] Wright M. S. Haft D. H. Harkins D. M. Perez F. Hujer K. M. Bajaksouzian S. (2014). New insights into dissemination and variation of the health care-associated pathogen *Acinetobacter baumannii* from genomic analysis. *MBio* 5:e963-13. 10.1128/mBio.00963-13 24449752 PMC3903280

[B51] Zankari E. Hasman H. Cosentino S. Vestergaard M. Rasmussen S. Lund O. (2012). Identification of acquired antimicrobial resistance genes. *J. Antimicrob Chemother.* 67 2640–2644. 10.1093/jac/dks261 22782487 PMC3468078

[B52] Zhou Y. Liang Y. Lynch K. H. Dennis J. J. Wishart D. S. (2011). PHAST: a fast phage search tool. *Nucleic Acids Res.* 39(Web Server issue), W347–W352. 10.1093/nar/gkr485 21672955 PMC3125810

[B53] Zordan S. Prenger-Berninghoff E. Weiss R. van der Reijden T. van den Broek P. Baljer G. (2011). Multidrug-resistant *Acinetobacter baumannii* in veterinary clinics, Germany. *Emerg. Infect. Dis.* 17 1751–1754. 10.3201/eid1709.101931 21888812 PMC3322069

[B54] Zurawski D. V. Thompson M. G. McQueary C. N. Matalka M. N. Sahl J. W. Craft D. W. (2012). Genome sequences of four divergent multidrug-resistant *Acinetobacter baumannii* strains isolated from patients with sepsis or osteomyelitis. *J. Bacteriol.* 194 1619–1620. 10.1128/JB.06749-11 22374953 PMC3294875

