# Supplementary material for: Virulent Epidemic Pneumonia in Sheep Caused by the Human Pathogen Acinetobacter baumannii
Source: Front Microbiol. 2018 Nov 6;9:2616. doi: 10.3389/fmicb.2018.02616 (PMC6232368; doi:10.3389/fmicb.2018.02616)
Supplement: Supplementary file 1 [file Table_1.DOCX]

Supplementary Table S1. RND (resistance-nodulation-division) family and MATE (multidrug and toxic compound extrusion) family efflux pumps in the genome of strain AbPK1.

| Gene product/function | Gene | Locus_tag in ATCC 17978 |
| --- | --- | --- |
| RND membrane fusion protein AdeA | *adeA* | A1S_1752/1751 |
| RND ATP-binding/permease pump protein AdeB | *adeb* | A1S_1750 |
| RND outer membrane protein AdeC | *adeC* | - |
| RND membrane fusion protein AdeF | *adeF* | A1S_2304 |
| RND ATP-binding/permease pump protein AdeG | *adeG* | A1S_2305 |
| RND outer membrane protein AdeH | *adeH* | A1S_2306 |
| RND membrane fusion protein AdeI | *adeI* | A1S_2735 |
| RND ATP-binding/permease pump protein AdeJ | *adeJ* | A1S_2736 |
| RND outer membrane protein AdeK | *adeK* | A1S_2737 |
| RND membrane fusion protein MacA | *macA* | A1S_0538/0537 |
| RND ATP-binding/permease pump protein MacB | *macB* | A1S_0536 |
| RND outer membrane protein MacC | *macC* | A1S_0535 |
| RND metal efflux membrane fusion protein CscA | *cscA* | A1S_3217 |
| RND ATP-binding/permease pump protein CscB | *cscB* | A1S_3218 |
| RND metal efflux outer membrane protein CscC | *cscC* | A1S_3217 |
| RND membrane fusion protein AcrA | *acrA* | A1S_2817 |
| RND ATP-binding/permease pump protein AcrB | *acrB* | A1S_2818 |
| RND membrane fusion protein | *acrA-like* | A1S_3447 |
| RND ATP-binding/permease pump protein | *acrB-like* | A1S_3446 |
| RND ATP-binding/permease pump protein | *adeB-like* | A1S_2660 |
| RND multidrug resistance efflux pump protein | *emrA* | A1S_1773 |
| RND multidrug resistance efflux pump protein | *emrB* | A1S_1772 |
| MATE family efflux pump | *norM* | A1S_3371 |
| MATE family efflux pump |  | A1S_3420 |
| MATE family efflux pump |  | A1S_0395 |
| Small multidrug resistance (SMR) family protein | *qacEΔ1-like* | A1S_2844 |
| Multidrug resistance transporter of Bcr/CflA family |  | A1S_2795 |
| Multidrug resistance transporter of Bcr/CflA family |  | A1S_2584 |
