# Supplementary material for: Virulent Epidemic Pneumonia in Sheep Caused by the Human Pathogen Acinetobacter baumannii
Source: Front Microbiol. 2018 Nov 6;9:2616. doi: 10.3389/fmicb.2018.02616 (PMC6232368; doi:10.3389/fmicb.2018.02616)
Supplement: Supplementary file 2 [file Data_Sheet_1.PDF]

# Figure S1

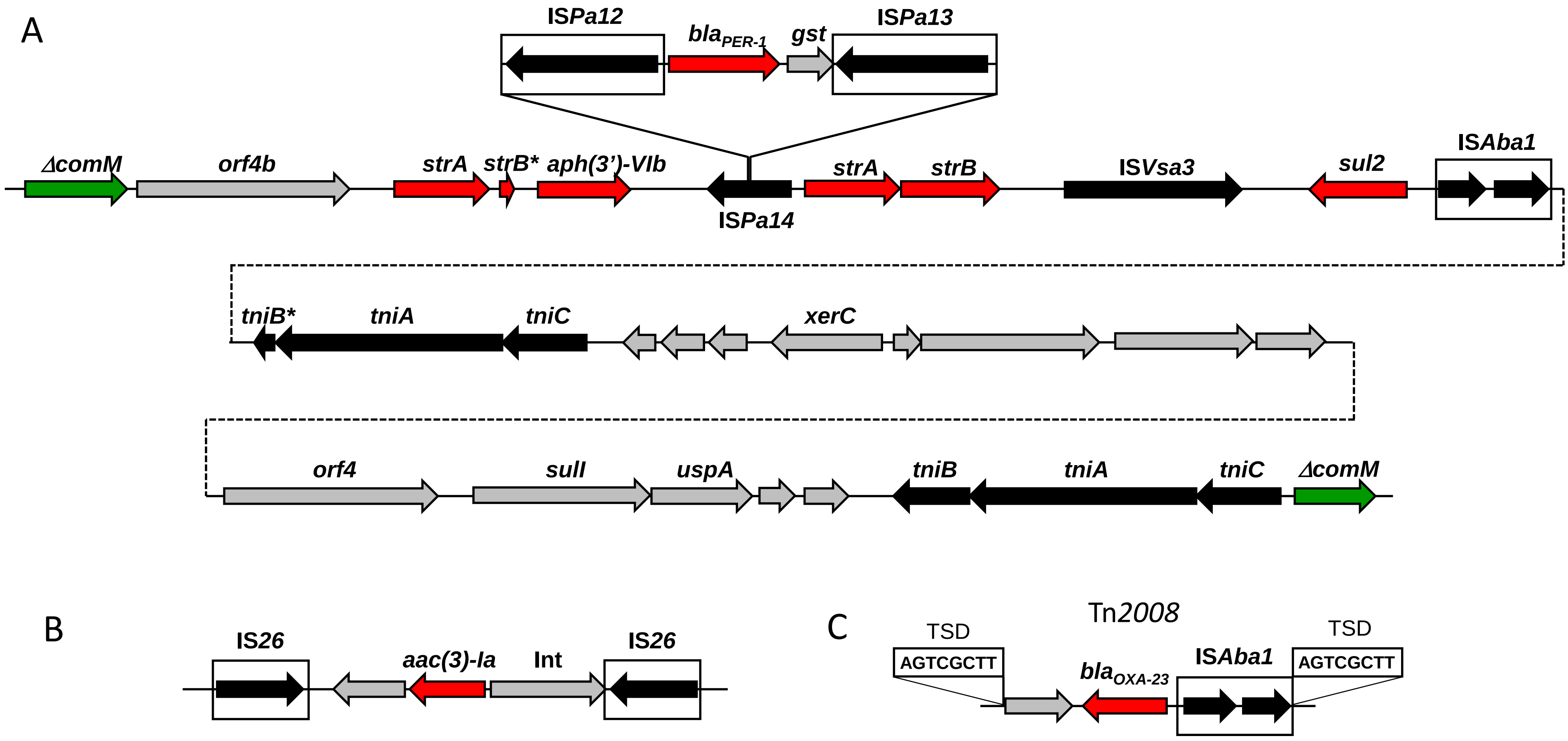


**Figure S1. Examples of antibiotic resistance genes associated with insertion sequence elements.** A) Resistance Island 1 (GI7) inserted into the *comM* gene containing resistance genes against aminoglycosides (*strA*, *strB*, *aph(3")-VIb*), β-lactams (*bla*$_{PER}$*-1*) and sulphonamides (*sul2*). B) Gentamicin-3'-N-acetyltransferase gene *aac(3)-Ia* flanked by inverted copies of IS*26*. C) Transposon Tn*2008* with *bla*$_{OXA}$*-23* was present as two copies on the bacterial chromosome and a third copy on plasmid pAbPK1b. TSD – target site duplication.
